# Supplementary material for: Up to 1 in 4 Veterans With Primary Biliary Cholangitis May Have Cirrhosis by the Time of Its Diagnosis
Source: Gastro Hep Adv. 2026 Apr 24;5(7):100983. doi: 10.1016/j.gastha.2026.100983 (PMC13218242; doi:10.1016/j.gastha.2026.100983)
Supplement: Supplementary Figure [file mmc1.pdf]

**Supplementary Figure 1. Number of overlapping patients identified across various PBC definitions evaluated**

| | **One ICD-9/10 and AMA positive (N = 1,002)** | **Two AMA positive (N = 2,065)** | **Two ICD-9/10 codes (N = 2,326)** | **>=1 inpatient or >=2 outpatient ICD-9/10 (N=2,647)** | **One ICD-9/10 Code (N=3,910)** |
|---|---|---|---|---|---|
| **One ICD-9/10 and AMA positive (N = 1,002)** | | | | | |
| **Two AMA positive (N = 2,065)** | # positive (row only): 1705<br>#positive (column only): 642<br># positive (overlap): 360 | | | | |
| **Two ICD-9/10 codes (N = 2,326)** | # positive (row only): 1523<br>#positive (column only): 199<br># positive (overlap): 803 | # positive (row only): 2021<br>#positive (column only):1760<br># positive (overlap): 305 | | | |
| **>=1 inpatient or >=2 outpatient ICD-9/10 (N = 2,647)** | # positive (row only): 1810<br>#positive (column only): 165<br># positive (overlap): 837 | # positive (row only): 2336<br>#positive (column only): 1754<br># positive (overlap): 311 | # positive (row only): 321<br>#positive (column only):0<br># positive (overlap): 2326 | | |
| **One ICD-9/10 Code (N = 3,910)** | # positive (row only): 2908<br>#positive (column only):0<br># positive (overlap): 1002 | # positive (row only): 3550<br>#positive (column only): 1705<br># positive (overlap): 360 | # positive (row only): 1584<br>#positive (column only):0<br># positive (overlap): 2326 | # positive (row only): 1263<br>#positive (column only): 0<br># positive (overlap): 2647 | |

Note: Positive (row only) are patients identified only with the row definition and not the column definition. Positive (column only) are patients identified only with the column definition and not the row definition. Positive (overlap) are patients who were identified with both definitions.
