# Supplementary material for: Up to 1 in 4 Veterans With Primary Biliary Cholangitis May Have Cirrhosis by the Time of Its Diagnosis
Source: Gastro Hep Adv. 2026 Apr 24;5(7):100983. doi: 10.1016/j.gastha.2026.100983 (PMC13218242; doi:10.1016/j.gastha.2026.100983)
Supplement: Extended PDF [file mmc2.pdf]

Gastro Hep Advances 2026;5:100983

ORIGINAL RESEARCH—CLINICAL

Up to 1 in 4 Veterans With Primary Biliary Cholangitis May Have Cirrhosis by the Time of Its Diagnosis

Robert J. Wong,[1,2] Zeyuan Yang,[1] and Ramsey Cheung[1,2]

[1]Gastroenterology Section, Veterans Affairs Palo Alto Healthcare System, Palo Alto, California; and [2]Division of Gastroenterology and Hepatology, Stanford University School of Medicine, Palo Alto, California

BACKGROUND AND AIMS: Existing studies suggest that up to half of individuals with primary biliary cholangitis (PBC) remain undiagnosed, contributing to lack of clarity on true prevalence of PBC. We aim to evaluate the prevalence of PBC among a national cohort of US veterans. **METHODS:** National Veterans Affairs data from January 1, 2010, to April 30, 2025, were evaluated to identify adults with PBC using a combination of International Classification of Diseases (ICD)-9/10 diagnostic codes (57.16, K74.3) and/or presence of positive antimitochondrial antibody. Prevalence of PBC (per 100,000 persons) and proportion of patients with cirrhosis at time of PBC diagnosis was evaluated by age, sex, and race/ethnicity. **RESULTS:** Among over 5.13 million veterans who had ≥1 annual health-care encounter, the prevalence of PBC ranged from 19.5 to 76.2 per 100,000 persons when using different combinations of ICD-9/10 codes and antimitochondrial antibody diagnostic criteria. Among most scenarios, PBC prevalence was highest among women, older individuals (age ≥60 years), and Hispanic, or Asian/Pacific Islanders. The proportion of patients with cirrhosis at time of PBC diagnosis ranged from 10.7% to 26.4%, with generally higher rates of cirrhosis in men vs women. **CONCLUSION:** Among a national cohort US veterans, the prevalence of PBC ranged from 19.5 to 76.2 per 100,000 persons when evaluating various diagnostic criteria. It is concerning that up to a quarter of patients may have already had cirrhosis at time of PBC diagnosis, emphasizing the importance of greater awareness of timely diagnosis and treatment of PBC.

Keywords: PBC; Veterans; Cholangitis; Corporate Data Warehouse; Cirrhosis

Introduction

Primary biliary cholangitis (PBC) is a progressive cholestatic liver disease associated with significant morbidity and mortality.[1–3] Delays in timely diagnosis and treatment contributes to continued liver disease progression to cirrhosis and subsequent liver-related mortality.[2–5] Existing studies suggest that as many as 50% of individuals with PBC may be undiagnosed, and the observation that up to 60% of individuals with PBC are asymptomatic may also have contributed to the delays in diagnosis.[5–8] Additional factors cited for potentially contributing to delays in PBC diagnosis and treatment include financial barriers, adherence to follow-up, severe decompensated disease at diagnosis, and lack of referral to specialists for further evaluation and treatment.[6] These gaps and delays in PBC diagnosis have contributed to the lack of clarity about the true prevalence of PBC in the United States.

A systematic review published in 2012 identified 24 articles eligible to assess the incidence or prevalence of PBC.[9] The majority of studies were from European regions, and only 2 studies evaluated PBC epidemiology in US cohorts.[10,11] Kim et al[11] evaluated data from the Rochester Epidemiology Project of Olmsted County, Minnesota from 1975 to 1995. A total of 46 patients with PBC were identified, which translated to a prevalence of 40.2 per 100,000 persons. Hurlburt et al[10] evaluated population-based data in Alaska from 1984 to 2000 to describe the epidemiology of autoimmune liver diseases. A total of 18 patients with PBC were identified among a cohort of 100,312 individuals, which translated to a prevalence of 16 per 100,000 persons. More recently, data from the Fibrotic Liver Disease Consortium evaluated data across 11 US health systems from 2003 to 2014 and reported PBC prevalence of 29.3 per 100,000 persons across the study period, with the annual prevalence increasing from 21.7 per 100,000 persons in 2006 to 39.2 per 100,000 persons in 2014.[12,13] Data from Levy et al[8] evaluated US administrative claims data using Komodo Healthcare Map and reported a PBC prevalence of 40.9 per 100,000 persons in 2021. However, the majority of these studies utilized diagnostic criteria based solely on International Classification of Diseases (ICD)-9/10 codes, which have inherent limitations. Specifically using ICD-9/10 codes alone to identify PBC is limited by low sensitivity and hence may underestimate the true prevalence of PBC, given suboptimal awareness of PBC, the observation that up to 60% of individuals with PBC are asymptomatic and thus may not receive appropriate

Abbreviations used in this paper: **AMA, antimitochondrial antibody; CDW, Corporate Data Warehouse; ICD, International Classification of Diseases; PBC, primary biliary cholangitis; SAS, Statistical Analysis System; VA, Veterans Affairs.**

Most current article

Published by Elsevier Inc. on behalf of American Gastroenterological Association Institute. This is an open access article under the CC BY license (http://creativecommons.org/licenses/by/4.0/).
2772-5723
https://doi.org/10.1016/j.gastha.2026.100983

workup and diagnosis, and previously noted multiple barriers to timely diagnosis and treatment in the PBC cascade of care.[3,5–7,12–14] Better understanding PBC epidemiology and elucidating potential gaps in PBC diagnosis are important to raise awareness among patients and providers so that individuals with underlying PBC can be diagnosed and linked to appropriate treatment in a timely manner to improve health outcomes and health-related quality of life. The current study utilizes national data from a longitudinal cohort of US veterans to provide estimates of PBC prevalence using both ICD-9/10 diagnostic codes and supporting laboratory data and to evaluate the severity of liver disease at time of PBC diagnosis.

Methods

Adults with PBC were identified using national longitudinal data from the Veterans Affairs (VA) Corporate Data Warehouse (CDW) from January 1, 2010 to April 30, 2025. The VA CDW captures data on over 7 million veterans who receive care in VA health centers and clinics in the United States. The CDW is a harmonized dataset that provides access to longitudinal laboratory data in addition to clinical encounters, clinical outcomes, and unique patient and clinical data at a granular level to be incorporated into data analyses. We performed a cross-sectional study to evaluate the prevalence of PBC using a combination of ICD-9/10 codes and laboratory results for antimitochondrial antibody (AMA). Given the aforementioned limitations of using ICD-9/10 diagnostic codes alone, we sought to explore different combinations of PBC diagnostic criteria that included ICD-9/10 diagnostic codes (57.16, K74.3) and/or presence of positive results for AMA. AMA is a highly disease-specific autoantibody detected in 90% to 95% of individuals with PBC and less than 1% of non-PBC controls.[1,3] We evaluated a series of PBC diagnostic criteria to provide a range of real-world prevalence estimates as follows: (1) presence of 1 PBC ICD-9/10 diagnostic code alone; (2) ≥1 inpatient or ≥2 outpatient PBC diagnostic codes; (3) ≥ 2 PBC diagnostic codes on separate clinical encounters; (4) ≥ 2 AMA positive results on separate clinical encounters; and (5) ≥1 PBC diagnostic code and ≥1 AMA positive result. PBC prevalence was calculated by dividing the number of individuals meeting aforementioned criteria with a denominator of individuals who had at least 1 health encounter each year of the study period. PBC prevalence (per 100,000 persons) was stratified by sex, age groups, and race/ethnicity. Comparisons of prevalence between groups were performed using the z-statistic based on standard equations.

We additionally evaluated the proportion of patients with cirrhosis at time of meeting PBC diagnostic criteria. Cirrhosis was identified using a combination of ICD-9/10 diagnostic codes using algorithms that have been previously used to identify cirrhosis in the VA CDW.[15,16] The proportion of patients with cirrhosis at the time of meeting PBC diagnostic criteria was stratified by sex, age groups, and race/ethnicity. Comparisons of the proportion of patients with cirrhosis between groups utilized chi-square testing. Statistical analyses were performed using structured query language and Statistical Analysis System (SAS) Studio 3.6 on SAS (version 9.4; SAS Institute Inc, Cary, NC). Statistical significance was met with 2-tailed P value <.05. This study was approved by the Stanford University institutional review board and VA Palo Alto Health Care System scientific research committee.

Results

Among over 5.13 million veterans who had at least 1 health encounter each year from January 1, 2010, to April 30, 2025, the prevalence of PBC ranged from 19.5 per 100,000 persons when using criteria requiring ≥1 PBC diagnostic code and ≥1 AMA positive result to 76.2 per 100,000 persons when using criteria requiring presence of 1 PBC ICD-9/10 diagnostic code (Figure 1). The 3 most similar PBC prevalence estimates were those based on ≥2 AMA positive results on different clinical encounters (40.2 per 100,000 persons), ≥2 PBC diagnostic codes on different

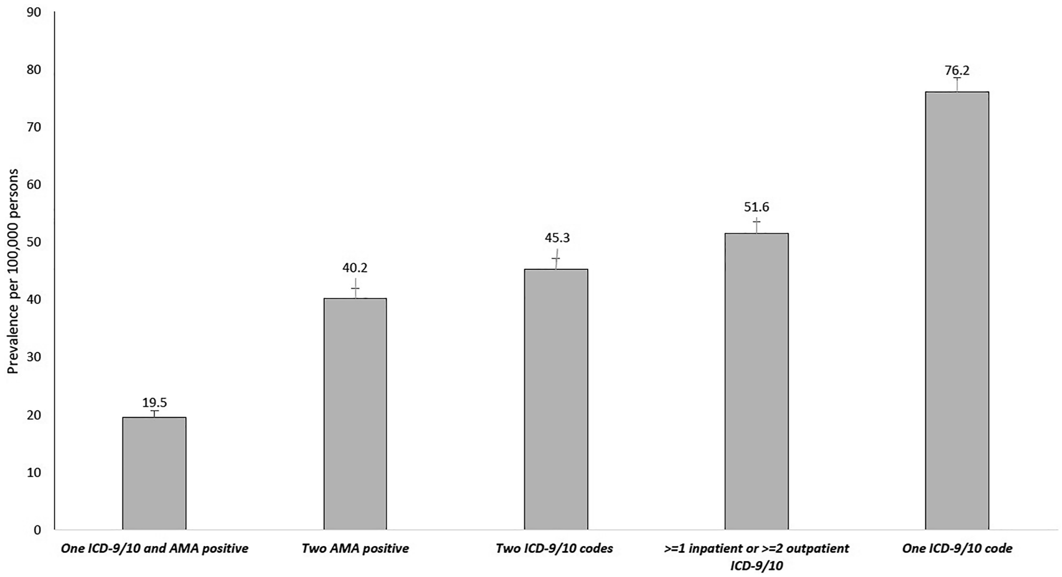

Figure 1. Prevalence of PBC among US veterans across different diagnostic criteria.

clinical encounters (45.3 per 100,000 persons), and ≥1 inpatient or ≥2 outpatient PBC diagnostic codes (51.6 per 100,000 persons). When evaluating across different definitions, the number of overlapping patients that were identified with multiple definitions ranged from 305 to 2647 (Supplementary Figure). For example, when comparing patients identified with 2 AMA positive results (n = 2065) or 2 ICD-9/10 codes (n = 2326), 305 patients were identified with both definitions.

The prevalence of PBC was significantly higher in women vs men across all diagnostic criteria, with the sex-specific differences greatest among the most specific criteria. For example, when using criteria requiring ≥1 PBC diagnostic code and ≥1 AMA positive result, PBC prevalence among women was more than 3 times higher than men (54.8 vs 15.8 per 100,000 persons, $P < .001$), whereas the gender gap was smaller (albeit still higher among women) with other criteria (eg, ≥2 AMA positive results: 69.6 per 100,000 persons in women vs 37.1 per 100,000 persons in men) (Table 1). Higher prevalences of PBC were seen with older age groups across most diagnostic criteria with the exception of the criteria based on ≥2 AMA results, where the highest prevalence was observed among individuals aged 40–59 years. When stratified by race/ethnicity, the majority of PBC patients identified were non-Hispanic White (Table 1). However, the prevalence of PBC was highest among Hispanics and Asian/Pacific Islanders.

Table 2 provides more detailed evaluation of sex-specific differences in PBC prevalence further stratified by age and race/ethnicity subgroups. While increasing age was associated with increasing PBC prevalence among both men and women, the gender gap became more prominent in older age groups. For example, when using the most strict criteria of requiring ≥1 PBC diagnostic code and ≥1 AMA positive result, PBC prevalence among adults aged 18–39 years was ∼4 times higher in women vs men (12.5 vs 3.2 per 100,000 persons, $P < .001$). However, among the oldest age group (age ≥60 years), PBC prevalence was more than 6 times higher in women vs men (134.4 vs 22.2, $P < .001$). Similar trends were seen across all PBC diagnostic criteria evaluated. When stratified by race/ethnicity, no consistently significant differences were observed likely due to relatively smaller sample sizes in some subgroups (Table 2).

Disease severity at time of diagnosis is a reflection of potential delays in timely diagnosis and treatment. The proportion of PBC patients with cirrhosis at the time of diagnosis ranged from 10.7% to 26.4% (Figure 2). PBC diagnostic criteria that were based on ICD-9/10 codes alone seemed to demonstrate higher proportions with cirrhosis compared to diagnostic criteria that incorporated AMA results. For example, the proportion of patients with cirrhosis at diagnosis was 23.1% and 26.4% when using diagnostic criteria requiring ≥2 PBC ICD-9/10 diagnostic codes on separate clinical encounters or 1 PBC diagnostic code, respectively, whereas the proportion with cirrhosis was 12.1% when using diagnostic criteria requiring ≥1 PBC diagnostic code and ≥1 AMA positive result. Across most PBC diagnostic criteria, the proportion with cirrhosis was significantly higher in men vs women (Figure 2). When stratified by sex, age, and race/ethnicity, the proportion with cirrhosis varied across subgroups (Table 3). When evaluating PBC diagnostic criteria based on ≥2 PBC diagnostic codes on separate clinical encounters, the highest proportion with cirrhosis was observed among young adults aged 18–39 years (men: 35.7%, 95% confidence interval [CI], 23.2–48.2; women: 41.4%, 95% CI, 23.5–59.3) and non-Hispanic Whites (men: 23.4%, 95% CI, 21.1–25.7; women: 28.2, 95% CI, 23.4–33.0) (Table 3).

Discussion

Among a national longitudinal cohort of over 5 million US veterans who are actively engaged in VA health care, we observed a range of real-world PBC prevalence estimates from 19.5 to 76.2 per 100,000 persons when exploring different combinations of PBC diagnostic criteria that incorporated the presence of ICD-9/10 diagnostic codes and/or positive AMA laboratory results. When using 1 ICD-9/10 code alone, PBC prevalence was 76.2 per 100,000 persons; however, when requiring the presence of both ICD-9/10 diagnostic code and a positive AMA result, the prevalence dropped to 19.5 per 100,000 persons. This is an interesting observation as it suggests a few key points. There were many patients with a PBC ICD-9/10 code who did not have an AMA positive result documented. For example, 3910 patients had at least 1 ICD-9/10 PBC diagnostic code and 1002 had both ICD-9/10 code and AMA positive, which suggests that only 25.6% of patients with a PBC diagnosis code had a corresponding AMA positive result documented. These variations in prevalence, when using different diagnostic criteria, also mirror the variations in PBC prevalence that has been reported in previous studies.[8,9] This may reflect underdiagnosis of PBC and potential misclassification bias when relying solely on ICD-9/10 diagnostic codes. On the other hand, it is also possible that this represent overdiagnosis—such that patients may be labeled at PBC with corresponding ICD-9/10 diagnosis codes based on other laboratory abnormalities or clinical factors without performing confirmatory AMA laboratory testing.

When we required the presence of ≥1 inpatient or ≥2 outpatient PBC diagnostic codes, PBC prevalence was 51.6 per 100,000 persons, which is similar to the prevalence of 40.9 per 100,000 persons reported in a recent administrative claims-based dataset study in the United States.[8] However, prevalence estimates that are based only on ICD-9/10 diagnostic codes are inherently biased and subject to underestimation, given that the presence of a documented diagnostic code requires that a provider is aware of, has evaluated for, and has made a clinical diagnosis of PBC. Prior studies have demonstrated that up to 50%–60% of

Table 1. Prevalence of Primary Biliary Cholangitis Across Different Diagnostic Criteria

Variables	One ICD-9/10 and AMA positive			Two AMA positive			Two ICD-9/10 codes		
	Prevalence per 100,000	95% CI	Frequency	Prevalence per 100,000	95% CI	Frequency	Prevalence per 100,000	95% CI	Frequency
Total	19.5	(18.3–20.7)	1002	40.2	(38.5–42.0)	2065	45.3	(43.5–47.2)	2326
Male	15.8	(14.6–16.9)	732	37.1	(35.4–38.9)	1722	38.7	(36.9–40.5)	1797
Female	54.8	(48.3–61.3)	270	69.6	(62.3–77.0)	343	107.4	(98.2–116.5)	529
Age 18–39 y	5.2	(3.7–6.7)	46	22.8	(19.6–25.9)	203	9.5	(7.5–11.6)	85
Age 40–59 y	17.8	(15.7–19.9)	280	48.8	(45.3–52.2)	768	36.3	(33.3–39.2)	571
Age ≥60 y	25.4	(23.5–27.3)	676	41.1	(38.6–43.5)	1094	62.7	(59.7–65.7)	1670
American Indian or Alaska Native	25.3	(8.8–41.8)	9	44.9	(22.9–66.9)	16	36.5	(16.7–56.3)	13
Asian or Pacific Islander	28.7	(17.4–39.9)	25	58.5	(42.4–74.5)	51	55.0	(39.5–70.6)	48
Black or African American	17.7	(14.8–20.5)	146	51.7	(46.8–56.6)	428	32.9	(29.0–36.8)	272
Hispanic	25.1	(19.8–30.4)	86	91.8	(81.6–101.9)	314	45.0	(37.9–52.1)	154
Non-Hispanic White	20.6	(19.0–22.1)	690	34.9	(32.9–36.9)	1171	50.7	(48.3–53.1)	1701

Variables	≥1 inpatient or ≥2 outpatient ICD-9/10			One ICD-9/10 code		
	Prevalence per 100,000	95% CI	Frequency	Prevalence per 100,000	95% CI	Frequency
Total	51.6	(49.6–53.5)	2647	76.2	(73.8–78.6)	3910
Male	45.0	(43.1–46.9)	2089	69.6	(67.2–72.0)	3228
Female	113.3	(103.9–122.7)	558	138.4	(128.0–148.8)	682
Age 18–39 y	10.7	(8.5–12.8)	95	15.3	(12.7–17.8)	136
Age 40–59 y	41.8	(38.6–45.0)	658	62.8	(58.9–66.7)	989
Age ≥60 y	71.1	(67.9–74.3)	1894	104.5	(100.6–108.4)	2785
American Indian or Alaska Native	42.1	(20.8–63.4)	15	64.6	(38.2–90.9)	23
Asian or Pacific Islander	64.2	(47.4–81.0)	56	89.4	(69.6–109.3)	78
Black or African American	38.9	(34.7–43.2)	322	61.0	(55.7–66.4)	505
Hispanic	52.3	(44.6–60.0)	179	77.4	(68.1–86.8)	265
Non-Hispanic White	57.1	(54.5–59.6)	1916	83.2	(80.1–86.3)	2792

Table 2. Prevalence of Primary Biliary Cholangitis Stratified by Males and Females

	Male			Female		
Variables	Prevalence per 100,000	95% CI	Frequency	Prevalence per 100,000	95% CI	Frequency
One ICD-9/10 and AMA positive						
Total	15.8	(14.6–16.9)	732	54.8	(48.3–61.3)	270
Age 18–39 y	3.2	(1.8–4.5)	22	12.5	(7.5–17.5)	24
Age 40–59 y	10.0	(8.3–11.7)	135	64.6	(54.1–75.2)	145
Age ≥60 y	22.2	(20.4–24.0)	575	134.4	(108.2–160.6)	101
American Indian or Alaska Native	15.8	(2.0–29.7)	5	98.3	(2.0–194.5)	4
Asian or Pacific Islander	17.0	(7.8–26.3)	13	109.9	(47.8–172.0)	12
Black or African American	12.4	(9.8–15.0)	88	49.0	(36.4–61.6)	58
Hispanic	21.7	(16.5–26.9)	67	56.4	(31.0–81.8)	19
Non-Hispanic White	16.7	(15.3–18.2)	523	72.8	(61.8–83.9)	167
Two AMA positive						
Total	37.1	(35.4–38.9)	1722	69.6	(62.3–77.0)	343
Age 18–39 y	23.0	(19.5–26.6)	161	21.8	(15.2–28.5)	42
Age 40–59 y	42.4	(38.9–45.8)	572	87.4	(75.1–99.6)	196
Age ≥60 y	38.2	(35.8–40.6)	989	139.7	(113.0–166.4)	105
American Indian or Alaska Native	28.5	(9.9–47.1)	9	172.0	(44.7–299.2)	7
Asian or Pacific Islander	45.9	(30.7–61.1)	35	146.5	(74.8–218.3)	16
Black or African American	50.0	(44.9–55.3)	355	61.7	(47.6–75.9)	73
Hispanic	92.0	(81.3–102.7)	284	89.1	(57.2–120.9)	30
Non-Hispanic White	31.1	(29.2–33.1)	973	86.3	(74.3–98.4)	198
Two ICD-9/10 codes						
Total	38.7	(36.9–40.5)	1797	107.4	(98.2–116.5)	529
Age 18–39 y	8.0	(5.9–10.1)	56	15.1	(9.6–20.6)	29
Age 40–59 y	21.3	(18.9–23.8)	288	126.2	(111.5–140.8)	283
Age ≥60 y	56.1	(53.2–59.0)	1453	288.7	(250.3–327.0)	217
American Indian or Alaska Native	25.4	(7.8–42.9)	8	122.8	(15.2–230.4)	5
Asian or Pacific Islander	44.6	(29.6–59.5)	34	128.2	(61.1–195.3)	14
Black or African American	24.5	(20.9–28.2)	174	82.8	(66.4–99.2)	98
Hispanic	37.3	(30.5–44.1)	115	115.8	(79.5–152.1)	39
Non-Hispanic White	43.4	(41.1–45.7)	1357	150.0	(134.2–165.8)	344
≥1 inpatient or ≥2 outpatient ICD-9/10						
Total	45.0	(43.1–46.9)	2089	113.3	(103.9–122.7)	558
Age 18–39 y	9.2	(6.9–11.4)	64	16.1	(10.5–21.8)	31
Age 40–59 y	26.7	(23.9–29.4)	360	132.8	(117.8–147.9)	298
Age ≥60 y	64.3	(61.2–67.4)	1665	304.6	(265.3–344.0)	229
American Indian or Alaska Native	28.5	(9.9–47.1)	9	147.4	(29.5–265.2)	6
Asian or Pacific Islander	55.1	(38.4–71.7)	42	128.2	(61.1–195.3)	14
Black or African American	30.6	(26.5–34.7)	217	88.8	(71.8–105.7)	105
Hispanic	44.7	(37.3–52.2)	138	121.7	(84.5–159.0)	41
Non-Hispanic White	49.7	(47.2–52.2)	1554	157.8	(141.6–174.1)	362
One ICD-9/10 code						
Total	69.6	(67.2–72.0)	3228	138.4	(128.0–148.8)	682
Age 18–39 y	13.7	(11.0–16.5)	96	20.8	(14.4–27.3)	40
Age 40–59 y	46.2	(42.6–49.8)	624	162.7	(146.0–179.4)	365
Age ≥60 y	96.8	(93.0–100.6)	2508	368.5	(325.2–411.8)	277
American Indian or Alaska Native	53.9	(28.3–79.5)	17	147.4	(29.5–265.2)	6
Asian or Pacific Islander	80.0	(59.9–100.0)	61	155.7	(81.7–229.6)	17
Black or African American	53.0	(47.7–58.4)	376	109.0	(90.2–127.8)	129
Hispanic	70.0	(60.7–79.3)	216	145.5	(104.8–186.2)	49
Non-Hispanic White	75.2	(72.2–78.2)	2352	191.8	(173.9–209.8)	440

patients with PBC are asymptomatic and may remain undiagnosed and unaware of having PBC.[2,5–7] Hence, the true prevalence of PBC must be higher than what has been reported in studies that rely only on ICD-9/10 diagnostic codes.

In our current cohort, PBC prevalence was higher in women vs men, which is consistent with prior epidemiological studies.[1–3,11,17,18] However, given the demographics of our cohort (VA population), our study identified a large proportion of men with PBC. We generally observed increasing prevalence with older age, which is also aligned with prior epidemiological studies in other US cohorts.[2,3,11,13] Among most of the criteria evaluated, the highest prevalence was seen

Table 3. Proportion of Patients With Advanced Liver Disease at the Time of Primary Biliary Cholangitis Diagnosis

Variables	Male Proportion	Male 95% CI	Male Frequency	Female Proportion	Female 95% CI	Female Frequency	Total Proportion	Total 95% CI	Total Frequency
One ICD-9/10 and AMA positive									
Total	15	(12.4–17.6)	110	4.1	(1.7–6.5)	11	12.1	(10.1–14.1)	121
Age 18–39 y	4.5	(0–13.1)	1	0	–	0	2.2	(0–6.5)	1
Age 40–59 y	11.9	(6.4–17.4)	16	3.4	(0.5–6.3)	5	7.5	(4.4–10.6)	21
Age ≥60 y	16.2	(13.2–19.2)	93	5.9	(1.3–10.5)	6	14.6	(11.9–17.3)	99
American Indian or Alaska Native	40	(0–82.9)	2	0	–	0	22.2	(0–49.3)	2
Asian or Pacific Islander	15.4	(0–35.0)	2	8.3	(0–23.9)	1	12	(0–24.7)	3
Black or African American	9.1	(3.1–15.1)	8	5.2	(0–10.9)	3	7.5	(3.2–11.8)	11
Hispanic	16.4	(7.5–25.3)	11	0	–	0	12.8	(5.7–19.9)	11
Non-Hispanic White	15.5	(12.4–18.6)	81	4.2	(1.2–7.2)	7	12.8	(10.3–15.3)	88
Two AMA positive									
Total	11.6	(10.1–13.1)	200	5.8	(3.3–8.3)	20	10.7	(9.4–12.0)	220
Age 18–39 y	3.1	(0.4–5.8)	5	4.8	(0–11.3)	2	3.4	(0.9–5.9)	7
Age 40–59 y	10.5	(8.0–13.0)	60	5.1	(2.0–8.2)	10	9.1	(7.1–11.1)	70
Age ≥60 y	13.7	(11.6–15.8)	135	7.6	(2.5–12.7)	8	13.1	(11.1–15.1)	143
American Indian or Alaska Native	0	N/A	0	0	N/A	0	0	N/A	0
Asian or Pacific Islander	5.7	(0–13.4)	2	6.3	(0–18.3)	1	5.9	(0–12.4)	3
Black or African American	9.9	(6.8–13.0)	35	4.1	(−0.4 to 8.6)	3	8.9	(6.2–11.6)	38
Hispanic	11.3	(7.6–15.0)	32	3.3	(−3.1 to 9.7)	1	10.5	(7.1–13.9)	33
Non-Hispanic White	12.3	(10.2–14.4)	120	6.6	(3.1–10.1)	13	11.4	(9.6–13.2)	133
Two ICD-9/10 codes									
Total	21.9	(20.0–23.8)	394	27	(23.2–30.8)	143	23.1	(21.4–24.8)	537
Age 18–39 y	35.7	(23.2–48.2)	20	41.4	(23.5–59.3)	12	37.6	(27.3–47.9)	32
Age 40–59 y	21.9	(17.1–26.7)	63	31.8	(26.4–37.2)	90	26.8	(23.2–30.4)	153
Age ≥60 y	21.4	(19.3–23.5)	311	18.9	(13.7–24.1)	41	21.1	(19.1–23.1)	352
American Indian or Alaska Native	25	(0–55.0)	2	60	(17.1–102.9)	3	38.5	(12.0–65.0)	5
Asian or Pacific Islander	17.6	(4.8–30.4)	6	14.3	(0–32.6)	2	16.7	(6.1–27.3)	8
Black or African American	15.5	(10.1–20.9)	27	23.5	(15.1–31.9)	23	18.4	(13.8–23.0)	50
Hispanic	16.5	(9.7–23.3)	19	23.1	(9.9–36.3)	9	18.2	(12.1–24.3)	28
Non-Hispanic White	23.4	(21.1–25.7)	317	28.2	(23.4–33.0)	97	24.3	(22.3–26.3)	414
≥1 inpatient or ≥2 outpatient ICD-9/10									
Total	25.3	(23.4–27.2)	529	25.4	(21.8–29.0)	142	25.3	(23.6–27.0)	671
Age 18–39 y	40.6	(28.6–52.6)	26	38.7	(21.6–55.8)	12	40	(30.1–49.9)	38
Age 40–59 y	27.2	(22.6–31.8)	98	30.9	(25.7–36.1)	92	28.9	(25.4–32.4)	190
Age ≥60 y	24.3	(22.2–26.4)	405	16.6	(11.8–21.4)	38	23.4	(21.5–25.3)	443
American Indian or Alaska Native	22.2	(0–49.3)	2	66.7	(29.0–104.4)	4	40	(15.2–64.8)	6
Asian or Pacific Islander	28.6	(14.9–42.3)	12	14.3	(0–32.6)	2	25	(13.7–36.3)	14
Black or African American	19.4	(14.1–24.7)	42	22.9	(14.9–30.9)	24	20.5	(16.1–24.9)	66
Hispanic	22.5	(15.5–29.5)	31	22	(9.3–34.7)	9	22.3	(16.2–28.4)	40
Non-Hispanic White	26.3	(24.1–28.5)	409	26.2	(21.7–30.7)	95	26.3	(24.3–28.3)	504
One ICD code									
Total	29.1	(27.5–30.7)	939	13.9	(11.3–16.5)	95	26.4	(25.0–27.8)	1034
Age 18–39 y	27.1	(18.2–36.0)	26	12.5	(2.3–22.7)	5	22.8	(15.7–29.9)	31
Age 40–59 y	37.2	(33.4–41.0)	232	13.4	(9.9–16.9)	49	28.4	(25.6–31.2)	281

Table 3. Continued

Variables	Male			Female			Total		
	Proportion	95% CI	Frequency	Proportion	95% CI	Frequency	Proportion	95% CI	Frequency
Age ≥60 y	27.2	(25.5–28.9)	681	14.8	(10.6–19.0)	41	25.9	(24.3–27.5)	722
American Indian or Alaska Native	35.3	(12.6–58.0)	6	16.7	(0–46.6)	1	30.4	(11.6–49.2)	7
Asian or Pacific Islander	32.8	(21.0–44.6)	20	17.6	(0–35.7)	3	29.5	(19.4–39.6)	23
Black or African American	30.1	(25.5–34.7)	113	11.6	(6.1–17.1)	15	25.3	(21.5–29.1)	128
Hispanic	32.4	(26.2–38.6)	70	14.3	(4.5–24.1)	7	29.1	(23.6–34.6)	77
Non-Hispanic White	28.7	(26.9–30.5)	676	13.6	(10.4–16.8)	60	26.4	(24.8–28.0)	736

in Hispanic and non-Hispanic White populations. Higher prevalence among Asian or Pacific Islanders were seen with some criteria, but the relatively small sample size among this ethnic group did not lend to significant differences in prevalence rates when compared to other groups.

Delays in PBC diagnosis can lead to continued liver disease progression and more severe disease at presentation. In our cohort, nearly 1 in 4 patients with PBC already had cirrhosis at the time of meeting PBC diagnostic criteria. This might reflect the observation that those with ICD-9/10 code-based criteria were more likely to be symptomatic or had advanced liver disease (eg, cirrhosis), which prompted the evaluation for liver disease etiology, leading to diagnosis of PBC. However, it is not clear why only 12.1% had cirrhosis among the cohort diagnosed with the stricter criteria of both ICD-9/10 code and positive AMA result. Across most criteria (with the exception of 2 ICD-9/10 codes), the proportion with cirrhosis was higher in men vs women. This is an interesting observation given that some studies have reported more severe disease progression and greater risk of liver-related mortality in men with PBC. For example, a prior study of 532 patients with PBC-related cirrhosis observed that men had an 80% higher risk of death or liver transplantation and more than 2 times higher risk of liver-related death.[19] A European study with over 3000 patients with PBC in Italy and Denmark also demonstrated that men had 2–3 times higher risk of all-cause mortality compared to women.[18] While it is not entirely clear why men with PBC have higher risk of disease progression and liver-related mortality, the "myth" that PBC is less common in men may contribute to delays in diagnosis, leading to more advanced disease at time of diagnosis, which is associated fewer options for successful treatment and worse outcomes.[3,6,20] Nevertheless, the observation that up to a quarter of PBC patients in our cohort may have already developed cirrhosis at the time of diagnosis is alarming and emphasizes the importance of raising awareness for early recognition and diagnosis, followed by prompt referral to specialty care and treatment. Since many patients with PBC are asymptomatic, it is likely that development of advanced liver disease such as cirrhosis prompts further workup and diagnosis of PBC.

The utilization of a national longitudinal cohort of over 5 million veterans who are actively engaged into care is a strength of our study. Furthermore, the utilization of both ICD-9/10 diagnostic codes as well as AMA laboratory results to identify PBC in such a large cohort distinguishes our study from existing smaller cohorts or administrative claims-based studies that rely on ICD codes alone. However, certain limitations should be acknowledged. While we utilized both ICD-9/10 diagnostic codes as well as AMA laboratory results, not all patients had recent alkaline phosphatase results to be incorporated into the definitions, which may have contributed to some degree of misclassification bias. However, our utilization of ICD-9/10 codes, in particular, are similar to prior studies using administrative claims datasets and allows comparison of prevalence

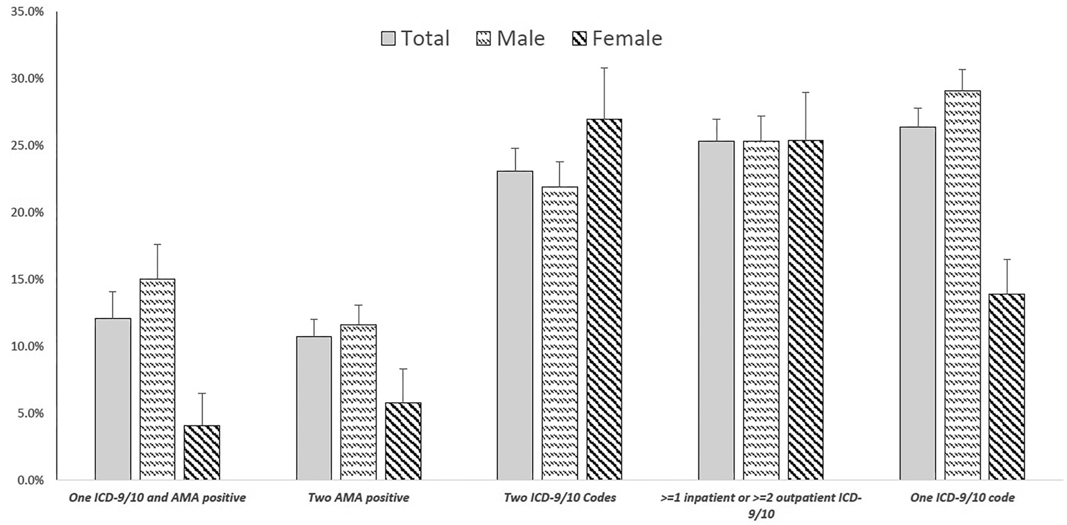

Figure 2. Proportion of patients with cirrhosis at the time of meeting PBC diagnostic criteria.

estimates from those studies with the current data. It is generally understood that prevalence of PBC is higher in women vs men, which was observed in our study. Yet, the predominantly male and older demographic of the VA population limits the generalizability of the findings to other populations. However, given the male predominance, our data do provide unique data to fill existing gaps in our understanding of PBC epidemiology among men, an understudied demographic in PBC. While we attempted to provide a broader range of real-world estimates of PBC prevalence that incorporate both ICD-9/10 codes and AMA laboratory results, we acknowledge that even with this approach, there likely remains a significant number of undiagnosed PBC patients who have not undergone AMA testing. While AMA is quite specific for the diagnosis of PBC, it is interesting that many patients in our cohort with ICD-9/10 diagnosis codes for PBC did not have a documented AMA positive result. It is possible that some of these patients may have received care and testing outside of VA health system. Another possibility is that these patients may have AMA negative PBC. Reviewing clinical notes for free text documentation of AMA results from outside labs was not feasible with the current dataset, but this could be a method applied to future studies to further improve accuracy of PBC diagnosis. The identification of cirrhosis was based on a combination of ICD-9/10 diagnostic codes using algorithms that have been previously used to identify cirrhosis in the VA CDW across liver disease etiologies.[15,16] However, there remains the possibility of misclassification bias that should be considered.

Conclusion

Among a national longitudinal cohort of US veterans, we observed a range of prevalence estimates for PBC ranging from 19.5 to 76.2 per 100,000 persons. Across different PBC diagnostic criteria, up to one-quarter of patients had already developed cirrhosis at time of diagnosis, emphasizing the need for improved efforts toward early recognition and diagnosis, followed by prompt referral to specialty care for monitoring and treatment.

Supplementary Materials

Material associated with this article can be found, in the online version, at https://doi:10.1016/j.gastha.2026.100983.

References

1. Kaplan MM, Gershwin ME. Primary biliary cirrhosis. N Engl J Med 2005;353:1261–1273.
2. Trivella J, John BV, Levy C. Primary biliary cholangitis: epidemiology, prognosis, and treatment. Hepatol Commun 2023;7:e0179.
3. Younossi ZM, Bernstein D, Shiffman ML, et al. Diagnosis and management of primary biliary cholangitis. Am J Gastroenterol 2019;114:48–63.
4. Gershwin ME, Selmi C, Worman HJ, et al. Risk factors and comorbidities in primary biliary cirrhosis: a controlled interview-based study of 1032 patients. Hepatology 2005;42:1194–1202.
5. Prince MI, Chetwynd A, Craig WL, et al. Asymptomatic primary biliary cirrhosis: clinical features, prognosis, and symptom progression in a large population based cohort. Gut 2004;53:865–870.
6. Shamaa O, Ahmed A, Rupp L, et al. Beyond the surface: unveiling hidden hurdles to primary biliary cholangitis care. Cureus 2024;16:e64753.
7. Garrido I, Liberal R, Cardoso MJ, et al. The impact of undiagnosed primary biliary cholangitis. Eur J Gastroenterol Hepatol 2021;33:e1027–e1031.
8. Levy C, Buchanan-Peart KA, MacEwan JP, et al. A nationwide study of primary biliary cholangitis prevalence, geographic distribution, and health care providers. Hepatol Commun 2025;9:e0677.

9. Boonstra K, Beuers U, Ponsioen CY. Epidemiology of primary sclerosing cholangitis and primary biliary cirrhosis: a systematic review. J Hepatol 2012;56:1181–1188.
10. Hurlburt KJ, McMahon BJ, Deubner H, et al. Prevalence of autoimmune liver disease in Alaska Natives. Am J Gastroenterol 2002;97:2402–2407.
11. Kim WR, Lindor KD, Locke GR 3rd, et al. Epidemiology and natural history of primary biliary cirrhosis in a US community. Gastroenterology 2000;119:1631–1636.
12. Lu M, Li J, Haller IV, et al. Factors associated with prevalence and treatment of primary biliary cholangitis in United States health systems. Clin Gastroenterol Hepatol 2018;16:1333–1341.e6.
13. Lu M, Zhou Y, Haller IV, et al. Increasing prevalence of primary biliary cholangitis and reduced mortality with treatment. Clin Gastroenterol Hepatol 2018;16:1342–1350.e1.
14. John BV, Bastaich D, Dahman B, et al. Identifying patients with primary biliary cholangitis and cirrhosis using administrative data in a national cohort. Pharmacoepidemiol Drug Saf 2024;33:e70013.
15. Wong RJ, Yang Z, Cheung R, et al. Impact of longitudinal alcohol use patterns on long-term risk of cirrhosis among US veterans with steatotic liver disease. Gastroenterology 2024;166:1156–1165.e4.
16. Kanwal F, Taylor TJ, Kramer JR, et al. Development, validation, and evaluation of a simple machine learning model to predict cirrhosis mortality. JAMA Netw Open 2020;3:e2023780.
17. Lindor KD, Bowlus CL, Boyer J, et al. Primary biliary cholangitis: 2018 practice guidance from the American Association for the Study of Liver diseases. Hepatology 2019;69:394–419.
18. Lleo A, Jepsen P, Morenghi E, et al. Evolving trends in female to male incidence and male mortality of primary biliary cholangitis. Sci Rep 2016;6:25906.
19. John BV, Aitcheson G, Schwartz KB, et al. Male sex is associated with higher rates of liver-related mortality in primary biliary cholangitis and cirrhosis. Hepatology 2021;74:879–891.
20. Shaker M, Mansour N, John BV. Primary biliary cholangitis in males: pathogenesis, clinical presentation, and prognosis. Clin Liver Dis 2022;26:643–655.

Received October 20, 2025. Accepted April 17, 2026.

Correspondence:
Address correspondence to: Robert J. Wong, MD, MS, Division of Gastroenterology and Hepatology, Veterans Affairs Palo Alto Healthcare System, Stanford University School of Medicine, 3801 Miranda Ave – GI-111, Palo Alto, California 94304. e-mail: Rwong123@stanford.edu.

Authors' Contributions:
Robert J. Wong: Study concept and design, acquisition of data, statistical analyses, drafting of the manuscript, study supervision, and guarantor of the article. Dr Robert J. Wong had full access to the data in the study and takes responsibility for the integrity of the data and accuracy of the data analysis. Zeyuan Yang: Acquisition of data and statistical analyses. All authors: Analysis and interpretation of the data and critical revision of the manuscript for important intellectual content. This material is the result of work supported with resources and the use of facilities at the Veterans Affairs Palo Alto Health Care System and the Veterans Affairs Corporate Data Warehouse. The content of this manuscript does not represent the views of the Veterans Affairs or the United States Government.

Conflicts of Interest:
This author discloses the following: Robert J. Wong: Research funding (to his institution) from Gilead Sciences, Exact Sciences, Theratechnologies, Durect Corporation, and Madrigal Pharmaceuticals, and serves as consultant (without compensation) for Gilead Sciences, Salix, and Mallinckrodt Pharmaceuticals. The remaining authors disclose no conflicts.

Funding:
Gilead Sciences (CO-US-986-7502).

Ethical Statement:
This study was approved by Stanford University Institutional Review Board and Veterans Affairs Palo Alto Healthcare System Scientific Research Committee.

Data Transparency Statement:
Data, analytic methods, and study materials utilized in this study can be available upon request from other researchers.

Reporting Guidelines:
STROBE.

Gastro Hep Advances, Volume 5

Supplemental information

Up to 1 in 4 Veterans With Primary Biliary Cholangitis May Have Cirrhosis by the Time of Its Diagnosis

Robert J. Wong, Zeyuan Yang, and Ramsey Cheung

Supplementary Figure 1. Number of overlapping patients identified across various PBC definitions evaluated

	**One ICD-9/10 and AMA positive (N = 1,002)**	**Two AMA positive (N = 2,065)**	**Two ICD-9/10 codes (N = 2,326)**	**>=1 inpatient or >=2 outpatient ICD-9/10 (N=2,647)**	**One ICD-9/10 Code (N=3,910)**
**One ICD-9/10 and AMA positive (N = 1,002)**					
**Two AMA positive (N = 2,065)**	# positive (row only): 1705<br>#positive (column only): 642<br># positive (overlap): 360				
**Two ICD-9/10 codes (N = 2,326)**	# positive (row only): 1523<br>#positive (column only): 199<br># positive (overlap): 803	# positive (row only): 2021<br>#positive (column only):1760<br># positive (overlap): 305			
**>=1 inpatient or >=2 outpatient ICD-9/10 (N = 2,647)**	# positive (row only): 1810<br>#positive (column only): 165<br># positive (overlap): 837	# positive (row only): 2336<br>#positive (column only): 1754<br># positive (overlap): 311	# positive (row only): 321<br>#positive (column only):0<br># positive (overlap): 2326		
**One ICD-9/10 Code (N = 3,910)**	# positive (row only): 2908<br>#positive (column only):0<br># positive (overlap): 1002	# positive (row only): 3550<br>#positive (column only): 1705<br># positive (overlap): 360	# positive (row only): 1584<br>#positive (column only):0<br># positive (overlap): 2326	# positive (row only): 1263<br>#positive (column only): 0<br># positive (overlap): 2647	

Note: Positive (row only) are patients identified only with the row definition and not the column definition. Positive (column only) are patients identified only with the column definition and not the row definition. Positive (overlap) are patients who were identified with both definitions.
